# Development of a pelvic musculoskeletal model based on statistical shape modelling for estimating sacroiliac and pubic joint reaction forces in females

**DOI:** 10.1007/s10237-026-02110-5

**Published:** 2026-08-05

**Authors:** Jie Chen, Patria A. Hume, Hannah E. Wyatt, Julie Choisne

**Affiliations:** 1 Auckland Bioengineering Institute, The University of Auckland, Auckland, New Zealand; 2 Faculty of Health and Environmental Science, Sports Performance Research Institute New Zealand (SPRINZ), Auckland University of Technology, Auckland, New Zealand; 3 Brain Health Research Institute (BHRI), Auckland University of Technology, Auckland, New Zealand; 4 Technology and Policy Lab - Law School, The University of Western Australia, Perth, Australia; 5 Sport, Health and Rehabilitation Research Cluster, Faculty of Health, The University of Canterbury, Christchurch, New Zealand; 6 Biomolecular Interaction Centre, University of Canterbury, Christchurch, New Zealand

**Keywords:** Pelvic ring, Sacroiliac joint, Pubic symphysis, Statistical shape model, Musculoskeletal model

## Abstract

The sacroiliac joints (SIJs) and pubic symphysis (PS) form a ‘pelvic ring’ that plays a critical role in load transfer between the spine and lower extremities. Direct in vivo measurement of pelvic joint loading remains challenging, and existing musculoskeletal models often simplify or exclude pubic joint contributions. This study developed a female pelvis musculoskeletal model incorporating both SIJs and PS, integrated with a personalisation framework including: (1) a pelvis shape-scaling workflow based on a female statistical shape model; and (2) an inertia estimation workflow using 3D full-body scans. Kinematic and kinetic data were collected from eight healthy female participants during bilateral standing, single-leg standing and walking. Compressive and superior–inferior shear loads at the SIJs and PS were estimated using inverse dynamics and static optimisation. The model produced physiologically plausible joint loads consistent with previous studies. During bilateral standing, pubic joint loads were minimal, and SIJ loads showed minor asymmetries. Single-leg standing induced SIJ tensile (1.9 N/kg), pubic compression (2.2 N/kg), and superior–inferior shearing at the support-side SIJ (4 N/kg). During walking, pubic joint loads were closely related to SIJ loading patterns and appeared to facilitate load transmission from the stance-side SIJ to the swing-side SIJ. This pelvis musculoskeletal model provides a feasible tool for investigating SIJ and PS reaction forces in females, enabling a more physiologically realistic assessment of pelvic joint biomechanics than previously available models. The proposed framework is adaptable to other populations and supports future research on pelvic pain mechanisms, pregnancy-related adaptations, and sex-specific musculoskeletal disorders.

**Supplementary Information:**

The online version contains supplementary material available at https://doi.org/10.1007/s10237-026-02110-5.

## Introduction

The pelvis comprises three bones (two hemipelves and sacrum) and three joints (two sacroiliac joints (SIJs) and the pubic symphysis (PS)) (Grechenig et al. 2021). The SIJ exhibits a complex anatomical structure, featuring numerous strong ligaments and irregular articular cartilage surfaces (an interlocking surface) that effectively transmit loads (Kiapour et al. 2020). The PS provides a strong midline connection between the pubic bones, together with the SIJs, forming the ‘pelvic ring’ structure (Becker et al. 2010).

The adult ‘pelvic ring’ is a relatively rigid structure, permitting only minimal motion at these articulations (Grechenig et al. 2021), while transmitting substantial moments across them (Ricci et al. 2018, 2020; Ries et al. 1989). The acetabulum, embedded within this ring (Grechenig et al. 2021; Reize et al. 2006; Krebs et al. 2009), conveys substantial loads from the hip joint towards the SIJ and the PS (Dalstra and Huiskes 1995). Thus, the ‘pelvic ring’ plays a central role in the loading transfer between the upper and lower body (Kiapour et al. 2020; Scholten et al. 1988). Under diverse and frequent loading conditions during daily activities, pain in the SIJ or pubic region is commonly reported and can affect a diverse group of individuals, including athletes, patients with traumatic pelvic injuries, and pregnant women (Kiapour et al. 2020; Becker et al. 2010; Sequeira 1986; Cheer and Pearce 2009; Ronchetti et al. 1976).

Compared to males, females are more likely to develop pelvic joint pain (Kiapour et al. 2020; Cohen 2005; Cohen et al. 2013), which may be related to sex differences in pelvic joint morphology and mobility that may cause extra loading (Becker et al. 2010; Brunner et al. 1976; Ebraheim and Biyani 2003; Tani et al. 2023). A finite element (FE) study reported that female SIJs exhibited greater stresses, loads, and ligament strains than male SIJs, which could be a possible reason for the higher incidence of SIJ pain and the pelvic stress fracture in females (Joukar et al. 1976). In addition, hormonal changes and biomechanical alterations during pregnancy further increase the risk of pelvic joint pain or even injury (Ronchetti et al. 1976; Culligan et al. 2002). Estimation of the joint reaction forces inside the pelvis is therefore helpful for understanding pain mechanisms, especially for females.

Direct in vivo measurements of internal joint loads within the pelvis remain challenging (Ricci et al. 2020), and current knowledge largely depends on in vitro experiments and FE modelling approaches (Joukar et al. 1976; Meissner et al. 1996; Eichenseer et al. 1976; Toyohara et al. 2020; Icke and Koebke 2014). Although FE models offer precise and compelling estimations of internal pelvic loads, they are typically personalised and require detailed imaging data, limiting their applicability for full-body dynamic movement analysis. When an approximate estimation of loads during functional activities is needed, musculoskeletal modelling offers a more efficient alternative to FE modelling. However, only a limited number of 3D musculoskeletal models are currently available that can estimate pelvic joint reaction forces (Dijke et al. 1999; Pel et al. 2008a; Goel and Svensson 1977).

One musculoskeletal pelvis model developed by Gilbert et al. (Dijke et al. 1999) assumed that the PS contributes minimally to load transmission between the spine and legs and set the pubic joint loading to zero (Dijke et al. 1999; Pel et al. 2008a, 2008b). However, this model was only ‘validated’ under bipedal standing, thereby overlooking PS loads that may be significant in certain single-leg postures/movements (Icke and Koebke 2014; Fabeck et al. 1994; MacAvoy et al. 1997; Vrahas et al. 1995; Goel et al. 1978). The model from Goel et al. was built on the ‘pelvic ring’ theory, which included the PS (Goel and Svensson 1977). However, due to its simplified structure, the PS in this model could only simulate linear tensile or compressive forces (Goel and Svensson 1977), although shear loads may be significant in asymmetric situations (Icke and Koebke 2014). Such limitations constrain our ability to understand the internal joint reaction forces of the pelvis during various functional activities.

Although musculoskeletal modelling is considerably more computationally efficient than FE modelling, the use of generic models inevitably sacrifices personalised accuracy, thereby limiting the validity of the results (Dijke et al. 1999; Bakke et al. 2023). For example, the default sacrum geometry in OpenSim models, such as the 2392 or 2354 models, contains limited anatomical detail, which may increase uncertainty in identifying anatomical landmarks or defining joint locations (Remus et al. 2023). Despite the higher prevalence of pelvic and musculoskeletal pain in female populations, female-specific musculoskeletal models are scarce, in terms of both generic models and personalised modelling workflows. Statistical shape modelling (SSM) offers a means to generate population-specific and individualised anatomical morphologies, and its integration into musculoskeletal modelling has demonstrated greater accuracy and robustness compared with traditional linear scaling approaches (Bakke et al. 2023; Zhang et al. 2016; Bakke and Besier 2020; Carman et al. 2024). Moreover, estimating personalised body segment inertial parameters from 3D full-body scans (typically under the assumption of uniform density) has gained increasing interest (Robert et al. 2017; Kudzia et al. 2022; Chen et al. 2026), as recent advances in low-cost 3D scanning technologies provide simple and accessible methods for acquiring body shape data.

Therefore, the present study aimed to develop an OpenSim-based pelvic musculoskeletal model together with a personalisation framework to enable more anatomically and mechanically realistic female pelvic model. This framework was intended to improve understanding of load distribution within the SIJs and PS, thereby providing a feasible tool for investigating the biomechanical mechanisms underlying sacroiliac and pubic pain in females. To achieve this aim, we: (1) developed a pelvis musculoskeletal model grounded in the ‘pelvic ring’ theory, and integrated it into a full-body model to estimate SIJ and PS joint reaction forces; (2) established a SSM-based workflow for pelvis personalised shape scaling, and a workflow for pelvis segment inertial parameter estimation using 3D full-body scans; and (3) examined the computed joint reaction forces across multiple activities in female participants and compared them with prior findings to assess physiological plausibility.

## Methods

### Participants information

Eight healthy females (30.3 ± 2.5 years old, 168.4 ± 6.8 cm, 70.6 ± 12.0 kg) participated in this study. Ethical approval was received from the AUT Ethics Committee (reference number 21/49). Informed written consent was obtained from all participants. No participants had any known neuromusculoskeletal disorders. Inclusion criteria were females aged 18 years and older, without any chronic or acute pain or any injury of the musculoskeletal system in the past six months prior to data collection.

### Data collection and post-processing

Each participant wore a form-fitting sports bra and shorts, and a swim cap on their head for 3D full-body scans obtained using a calibrated Anthroscan body scanner (Human Solution, Kaiserslautern, Germany). The system employs four eye-safe lasers and eight cameras, capturing up to 300 data points per cm2 as a 3D point cloud through optical triangulation. Participants were briefed and positioned following the standard scanning posture: standing upright with feet placed on the platform markers (approximately 30 cm apart), arms slightly bent at the elbows and held away from the body, and the head facing forward. During the approximately 10-s scan, lung capacity was standardised by instructing participants to exhale and refrain from breathing in.

**Fig. 1:**
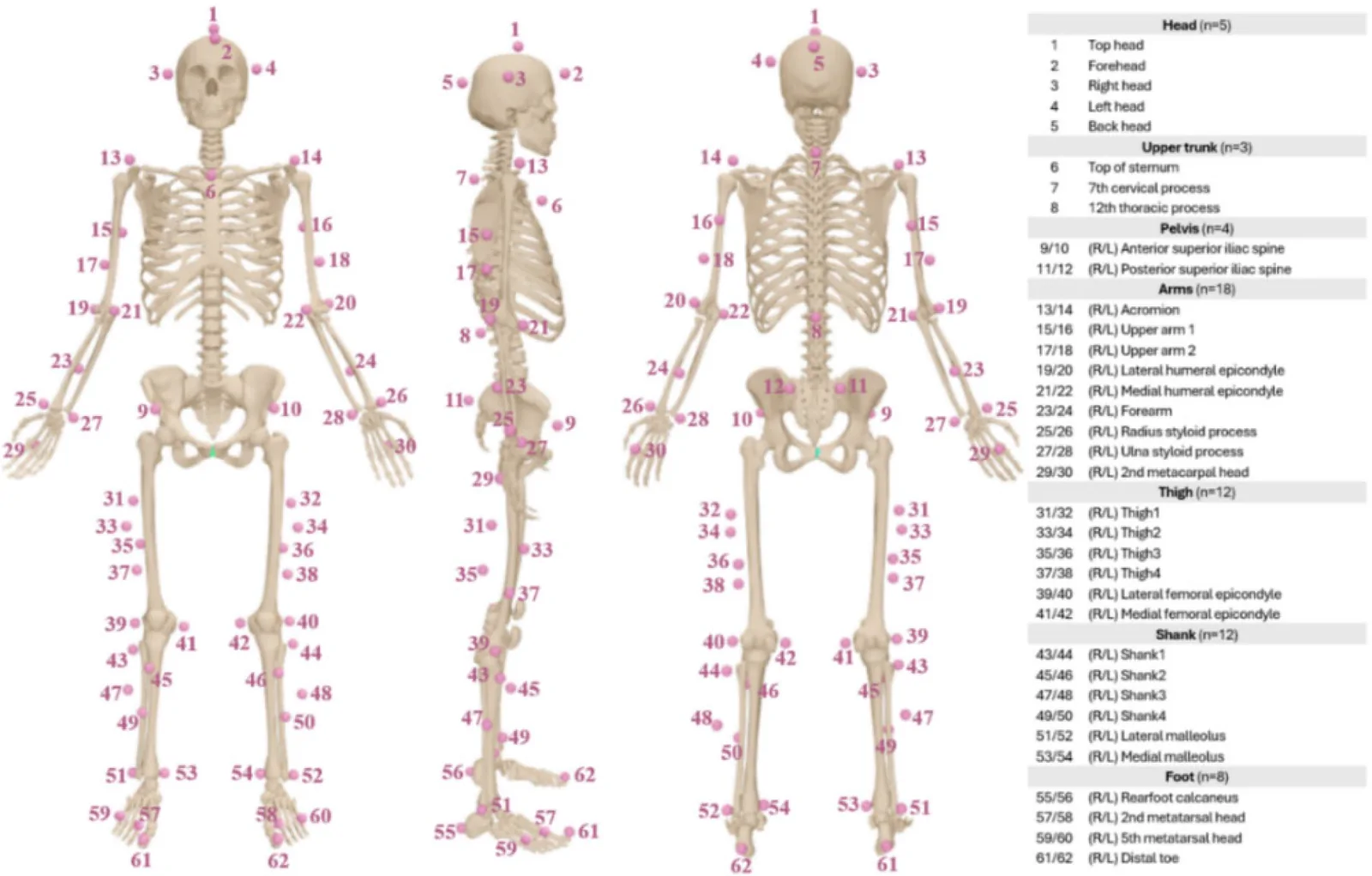
Marker placement locations for all participants

After the full-body scan, 62 retroreflective markers were placed on participants using hypoallergenic double-sided tape (Fig. 1). Markers’ trajectories were recorded at 200 Hz using the 8-camera Vicon optical motion capture system (Oxford Metrics Ltd., Oxford, UK). Two ground-embedded force plates (Bertec, Columbus, Ohio, USA) measured ground reaction forces at 1000 Hz. Participants performed two bilateral standing trials for 10 s with a foot on each force plate, feet shoulder width apart and hands on their hips, two right-leg standing trials for 10 s with hands on their hips, and ten walking trials at their self-selected speed.

All 3D scans were output as.obj files and checked for quality (absence of artefacts). Scans containing small holes or gaps were further inspected and smoothly filled in Meshmixer version 3.5.0 (Autodesk, San Rafael, CA, USA). Marker trajectory and ground reaction force data were synchronised and reconstructed in Vicon Nexus version 2.16, and then processed using a fourth-order Butterworth low-pass filter with a cutoff frequency of 10 Hz in MATLAB R2022b (MathWorks Inc., Natick, MA, USA).

### Pelvis statistical shape modelling

A statistical shape model (SSM) of the female pelvis was built to generate pelvis musculoskeletal models with personalised bony geometry for the eight study participants. The SSM training dataset consisted of 150 CT scans from a separate forensic population of female individuals (age: 34.2 ± 8.4y [20–49], height: 163.3 ± 7.9cm [155–195], body mass: 71.5 ± 20.4kg [43–166]) provided by the Victorian Institute of Forensic Medicine (VIFM, Melbourne, Australia) under ethics approval 2023-Choisne-1143–2385/3 from the VIFM Ethics Committee and AH24671 from the Auckland Health Research Ethics Committee was used to reconstruct the pelvic bone surface. The eight participants mentioned in 2.1 Participants were not included in the SSM training dataset.

**Fig. 2:**
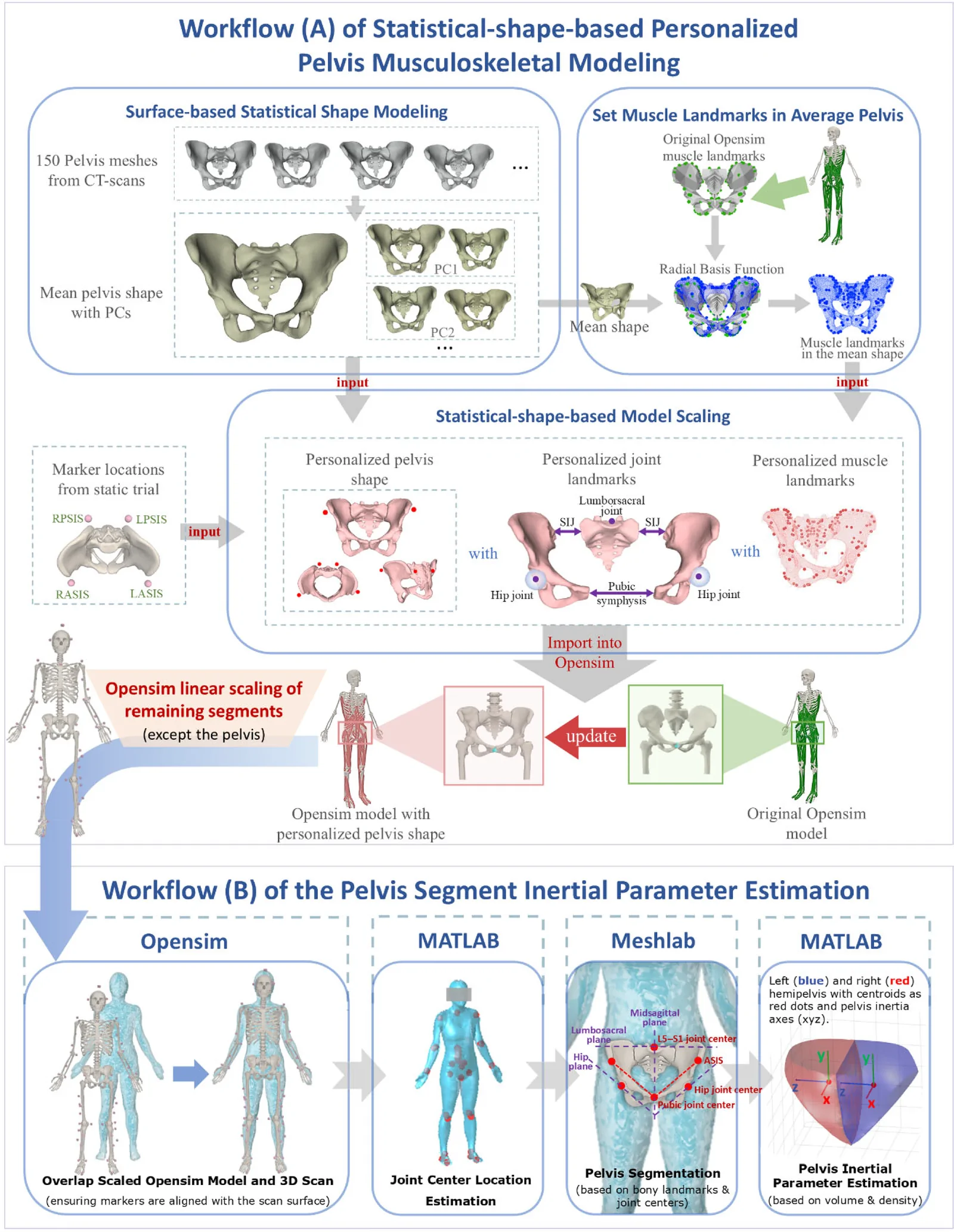
Workflow (A) of statistical shape-based personalised female pelvic musculoskeletal model and workflow (B) of the pelvis segment inertial parameter estimation based on 3D full-body scans. LASIS: left anterior superior iliac spines; RASIS: right anterior superior iliac spines; LPSIS: left posterior superior iliac spines; RPSIS: right posterior superior iliac spines; SIJ: sacroiliac joint

A workflow was developed based on the GIAS3 package (Zhang et al. 2016, 2018; Zhang and Besier 2017), to build the SSM of the pelvis (Fig. 2, workflow A). First, the pelvic bone (both hemipelvis and the sacrum) reconstructions were performed manually using Mimics version 23 (Materialise, Leuven, Belgium). Second, each of the pelvis meshes in the dataset was non-rigidly registered, using radial basis functions (RBFs), to a random pelvis template mesh to achieve nodal correspondence between the meshes (Zhang et al. 2018). Registration was performed using an iterative non-uniform Gaussian RBF framework, in which RBF centres (knots) were automatically added in regions with the largest residual registration errors to establish nodal correspondence between meshes. A maximum of 500 and 1000 knots were used in the coarse and fine registration stages, respectively, with convergence tolerances of 10^⁻1^ and 10^⁻3^ and a maximum of 20 iterations per stage. The mean registration root mean square error (RMSE) across the 150 training pelvises was 2.06 ± 0.39 mm, indicating good geometric correspondence between the registered meshes and the original segmented geometries. Third, we performed rigid alignment to remove rotational and translational variations, by aligning all bones to the template mesh using the centre of mass. The final step was to perform a principal component analysis (PCA) on the aligned meshes to generate the mean mesh (41,676 vertices and 83,420 triangles) and the principal components of variation in the dataset.

Bony landmarks on the mean pelvis mesh (the left and right anterior superior iliac spines (ASIS), the left and right posterior superior iliac spines (PSIS), the vertices of joint contact surfaces of lumbosacral, sacroiliac, and pubic joints, and the acetabular vertices (Appendix A1 in Supplementary Material)) were identified to define the coordinate system of the pelvis and the pelvis-related joints. Using a custom Python script (Carman et al. 2022, 2023), the mean pelvis mesh was aligned to the ISB-recommended pelvic coordinate system (Wu et al. 2002) based on the left and right ASIS and PSIS landmarks, with the origin located at the midpoint between the two ASIS. Specifically, a two-stage procedure was used to avoid circular dependency between landmark definition and coordinate system construction. First, initial landmark regions (LASIS, RASIS, LPSIS, and RPSIS) were manually selected as vertex groups to provide a coarse initialisation of pelvic orientation. Using this initial alignment, the ASIS and PSIS landmarks were then refined within each local vertex group by identifying coordinate extrema (i.e. maximum X-coordinate for ASIS and minimum X-coordinate for PSIS after transformation into the provisional coordinate system). The pelvic coordinate system was subsequently updated based on the refined landmarks, and this process was iteratively repeated until convergence.

Pelvis-related muscle landmarks were also identified on the mean pelvis mesh: First, pelvis muscle path points were extracted from an OpenSim model (Raabe’s full-body model (Raabe and Chaudhari 2016)) as 3D coordinates (.ply file). The OpenSim rough pelvis mesh was first remeshed (~ 80,000 triangles) in MeshLab and was then non-rigidly registered using RBF to the SSM produced mean pelvic mesh, resulting in an intermediate mesh A with the shape of the OpenSim pelvis and the mesh corresponding to the mean pelvis. Mesh A was subsequently rigidly aligned to the original OpenSim pelvis mesh by the centre of mass. For each exported OpenSim muscle landmark position, the nearest vertex on mesh A was identified, and the corresponding vertex index was recorded as the muscle landmarks on the mean pelvis template.

The mean pelvis shape was then used as the template for a second round of registrations of all individual meshes. Principal components (PCs) describing shape variation and the mean pelvis shape were computed from the second-round PCA.

Personalised pelvic geometry was reconstructed by fitting the SSM to 3D marker coordinates (ASIS and PSIS landmarks) obtained from the participants’ static trial, with constraints applied to preserve anatomical realism (Zhang et al. 2016). Reflective marker diameters and skin/soft tissue thickness (5 mm) were considered to correct for surface-to-bone offsets (Zhang et al. 2016). Reconstruction used the first eight PCs, capturing more than 85% of total pelvis shape variation (Xiang et al. 2024).

### Model articulation

Pelvic joint coordinate systems were determined from the bony landmarks derived from the personalised pelvis geometry obtained through SSM-based scaling. Joint centres for the lumbosacral, sacroiliac, and pubic joints were defined as the barycentre of their respective articulating regions (Appendix A1 in Supplementary Material): the L5-S1 lower endplates area for the lumbosacral joint, the combined sacral and iliac auricular surfaces for the SIJs (Scholten et al. 1988; Goel and Svensson 1977), and the opposing symphyseal joint surfaces of the left and right pubic bones for the PS (Becker et al. 2010). The right (or left) hip joint centre was obtained by fitting a sphere to the acetabular region of the pelvis (Appendix A1 in Supplementary Material). The origins of the hip, lumbosacral, sacroiliac, and pubic joint coordinate systems were positioned at their respective joint centres.

#### Hip and lumbosacral joint articulation

For the hip and lumbosacral joints, since only the pelvic geometry was modified, it was necessary to redefine the parent body coordinate system of their expression frames based on the updated pelvis. The orientation of the hip joint coordinate system on each hemipelvis was aligned with the ISB-recommended pelvis coordinate system described by Wu et al. (Wu et al. 2002). For the lumbosacral joint, a plane was fit to the L5-S1 articulating surface using a least-squares approach that minimised the sum of squared perpendicular distances from all articular vertices to the plane. The Y-axis of the lumbosacral joint was defined as the line passing through the lumbosacral joint centre and perpendicular to this plane, oriented superiorly (Chen et al. 2025). The X-axis was defined as the line lying within both the fitted articular plane and the sagittal plane of the pelvis, oriented anteriorly. The Z-axis was determined as the cross product of the X- and Y-axes, oriented to the right according to the right-hand rule.

#### Sacroiliac joints and pubic symphysis joint articulation

When establishing a joint coordinate system in OpenSim, two distinct aspects must be taken into consideration. The first is the joint expression coordinate system (the parent/child body coordinate system in OpenSim), which determines how joint forces and moments are expressed. The joint expression coordinate system only affects how calculated joint resultant forces are resolved onto the coordinate axes for reporting and interpretation. It does not influence the underlying joint mechanics, kinematics, or load transmission within the model. The second is the functional coordinate system, which defines the kinematic constraints and relative motion between adjacent segments, influencing the resulting joint reaction forces.

**Fig. 3:**
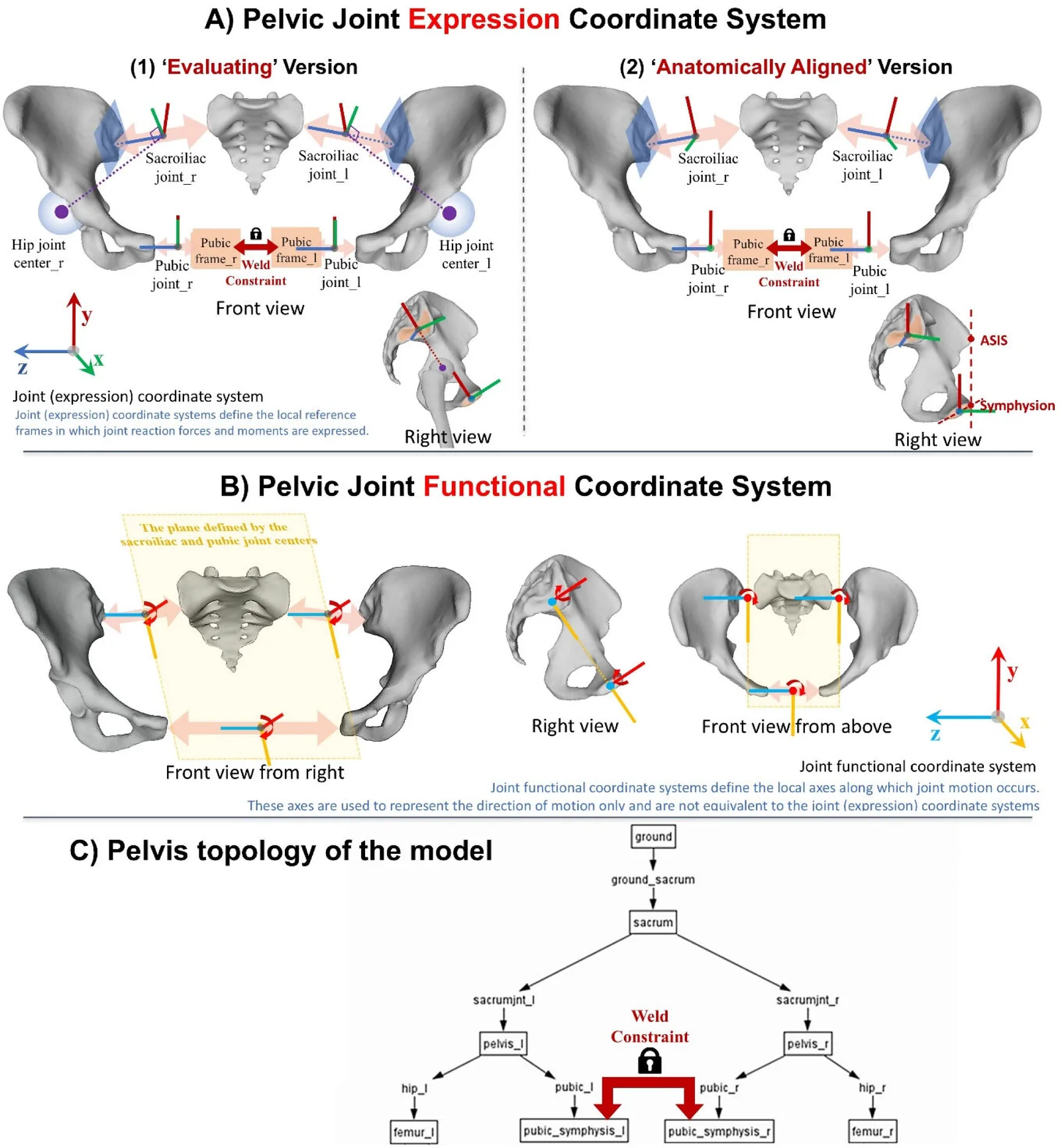
Schematic illustration of (A) the pelvic joint expression coordinate systems, including (1) the ‘evaluating’ version and (2) the ‘anatomically aligned’ version; (B) the definition of the pelvic joint functional coordinate system; and (C) the pelvis topology of the OpenSim model

Substantial discrepancies exist across modelling and in vivo/in vitro studies regarding the definition of SIJ and PS joint expression coordinate systems (Scholten et al. 1988; Meissner et al. 1996; Pel et al. 2008a). To enhance comparability and interpretability of the model outputs, two versions of the pelvic joint expression coordinate systems were established: (1) an ‘evaluating’ version (Fig. 3A-1), aligned with previous studies reporting pelvic joint loads (Scholten et al. 1988; Meissner et al. 1996), and (2) an ‘anatomically aligned’ version (Fig. 3A-2), whose axes more closely approximated the anatomical orientation of the pelvis in an upright posture (Reynold et al. 1982; Kai et al. 2014), to facilitate biomechanical interpretation. Both expression coordinate system versions shared the same pelvic joint functional coordinate systems and therefore differed only in how the resulting forces were expressed and reported.

(1) Pelvic ‘evaluating’ joint expression coordinate system definition.

Because the pelvis is naturally anteriorly tilted in the human standard anatomical position, some previous studies defined the anterior–posterior and superior–inferior directions of the sacroiliac (Scholten et al. 1988) and pubic joints (Meissner et al. 1996; Walheim et al. 1984) in ways that resulted in joint axes that were inclined relative to the pelvis anatomical coordinate system. However, these coordinate systems were not derived from anatomical landmarks and were described ambiguously. Therefore, in the present study, we reconstructed an ‘evaluating’ version of pelvic joint coordinate systems based on anatomical landmarks to approximate the conventions used in previous studies (Fig. 3A-1). This coordinate system was therefore implemented to facilitate direct comparison with previously reported pelvic joint loading studies.

Since the present study focused on SIJ compressive forces rather than nutation motion, the SIJ physiological coordinate system recommended by Van et al. was not directly implemented (Van Hauwermeiren et al. 2019). Instead, the coxal coordinate system, as described by Van et al. (Van Hauwermeiren et al. 2019), with the Z-axis perpendicular to the SIJ surface, was adopted to express SIJ reaction forces. Specifically, the Z-axis was defined as perpendicular to the plane of the SIJ surface and passing through the SIJ origin (O_SIJ_), oriented right (Fig. 3A-1). A temporary axis (Tc) connects O_SIJ_ to the hip joint centre. The X-axis is defined as the common perpendicular between the Z-axis and Tc through O_SIJ_, oriented anteriorly. Finally, the Y-axis is constructed perpendicular to both the X- and Z-axes, also passing through O_SIJ_ and oriented superiorly.

For the pubic joint expression coordinate system, the articular surface (the X–Y plane) corresponded to the X–Y plane in the pelvis coordinate system, with the Z-axis defined as perpendicular to it (Fig. 3A-1). The superior–inferior direction (Y-axis) was defined along the major axis of the pubic articular surface (Walheim et al. 1984). The joint’s major axis was identified within the Y–Z plane as the line passing through the pubic symphysis (PS) origin (O_PS_) and connecting the two farthest points on the joint contact surface. The X-axis was then constructed perpendicular to both the Y- and Z-axes, also passing through O_PS_.

(2) Pelvic ‘anatomically aligned’ joint expression coordinate system definition.

For the ‘anatomically aligned’ version, the pubic joint expression coordinate system was defined at the pubic joint centre and oriented according to an anatomical pelvic coordinate system constructed using the right and left ASISs and the pubic symphysion landmarks (midpoint between the left and right superior poles of the pubic symphysis, Fig. 3A-2) (Reynold et al. 1982). These three landmarks define the Y–Z plane. The Z-axis was defined as the line passing from the left ASIS to the right ASIS, oriented right. A line perpendicular to this Z-axis and passing through the symphysion defined the Y-axis, oriented superiorly (Fig. 3A-2). The X-axis was then constructed as the axis perpendicular to the Y–Z plane according to the right-hand rule, oriented anteriorly.

For the SIJ expression coordinate system, the Z-axis was defined as perpendicular to the best-fitted plane of the SIJ surface on the sacrum and passing through the SIJ origin (O_SIJ_), oriented right (Fig. 3A-2). The intersection between the SIJ surface plane and the pelvis anatomical Y–Z plane passing through the O_SIJ_ defined the Y-axis, oriented superiorly (Fig. 3A-2). The X-axis was constructed as the cross product of the Y- and Z-axes, oriented anteriorly.

(3) Pelvic joint functional coordinate system definition.

Both pelvic joint expression coordinate system versions (‘evaluating’ and ‘anatomically aligned’) shared the same pelvic joint functional coordinate systems. Although the SIJs and the PS exhibit small mobility, their range of motion is extremely limited (a few millimetres or degrees) and cannot be reliably captured by skin markers due to soft tissue artefacts (Kiapour et al. 2020; Becker et al. 2010; Toyohara et al. 2020; Pool-Goudzwaard et al. 2004). Therefore, consistent with previous pelvic musculoskeletal modelling studies (Raabe and Chaudhari 2016; Hamner et al. 2010), the pelvic ring was represented as a highly constrained articulated structure.

Although weld joints would provide a completely rigid representation of the pelvic ring, preliminary testing showed that modelling the sacroiliac joints as weld joints eliminated the joint reaction forces at the pubic symphysis. Therefore, hinge joints were selected as a practical compromise that preserved load transfer throughout the pelvic ring while maintaining a highly constrained articulation. The two SIJs and two pubic joints were modelled as hinge joints, permitting joint rotation about the axis perpendicular to the plane defined by the sacroiliac and pubic articulations (Fig. 3B). In this configuration, all pelvic joints lie within the same plane (the Y–Z plane of the joint functional coordinate systems), with adjacent segments allowed to rotate about axes perpendicular to this plane (the X-axes of the joint functional coordinate systems). This configuration minimises unintended internal pelvic motion during OpenSim simulations while preserving the transmission of full three-dimensional joint reaction forces throughout the pelvic ring.

### Muscle attachment and path

For muscle ‘attached’ points located on the pelvis surface, their new positions were obtained directly from the corresponding muscle landmarks on the SSM-scaled pelvis. For muscle via points (i.e. intermediate path points of the gluteal and psoas muscles) that do not lie on the bone surface, their positions were updated following the scaling approach implemented in OpenSim. Specifically, the coordinates of the via points relative to the pelvis were scaled according to the ratio between the participant-specific and generic pelvic widths, defined as the sum of the ASIS and PSIS widths.

Finally, the reconstructed pelvis was partitioned into the left and right hemipelves and sacrum and incorporated into the original OpenSim model together with the updated joint definitions and muscle attachments.

### OpenSim pelvis model development

To estimate the pelvic joint reaction forces during simulation, it was essential to incorporate detailed muscles surrounding the pelvis (Dijke et al. 1999). Thus, the new pelvic model was developed based on Raabe’s full-body model (Raabe and Chaudhari 2016), composed of a muscle-detailed spine model (Christophy et al. 2012) and lower-limb model (Hamner et al. 2010).

The sacrum segment was linked to the ground frame (Fig. 3C) and connected with the right and left hemipelves with two SIJs. To ensure pelvic symmetry and facilitate the interpretation of results, we introduced two pubic frames (pubic frame_r&l, Fig. 3A), each connected to the corresponding hemipelvis through a pubic joint (Fig. 3A). A weld constraint was then applied between the two pubic frames to form the closed pelvic ring (Fig. 3A) (Akhavanfar et al. 2022).

### Pelvis inertial parameter estimation

Following SSM-based scaling of the pelvis, all remaining model segments of each participant were linearly scaled in OpenSim to match each participant’s anthropometry (Delp et al. 2007). Virtual marker positions were simultaneously adjusted to align with experimental data during the Scaling process (Kai et al. 2014). The scaled OpenSim model was then repositioned and repostured to ensure its virtual markers aligned with the surface of the participant’s full-body 3D surface scan (.obj file) imported in OpenSim (Fig. 2, workflow B).

In this model posture, the locations of relevant anatomical landmarks and joint centres (bilateral ASIS, L5-S1, pubic joint, and bilateral hip centres) were computed and exported as a.ply file using MATLAB R2022b (Fig. 2, workflow B). These landmarks were then imported into MeshLab as references for segmenting the participant’s full-body surface scan. The scan was segmented using four anatomically defined planes to isolate the left and right hemipelves (Fig. 2, workflow B): 1. Lumbosacral plane: a transverse plane passing through the L5-S1 joint centre and extending horizontally across the torso. 2. Hip plane (right and left): transverse planes passing through each hip joint centre and parallel to the midsagittal plane defined by the ipsilateral ASIS landmark and the pubic joint centre. 3. Midsagittal plane: a vertical plane passing through the L5-S1 joint centre and the pubic joint centre. Each segmented hemipelvis region was filled and remeshed as a new, closed, triangular mesh (.obj file). Due to the irregular geometry and relatively small volume of the sacrum, the sacrum was not segmented separately.

Assuming uniform density of the whole pelvis (1020 kg/m^3^ (Pearsall et al. 1996)), the inertial parameters of each hemipelvis region (mass, centre of mass, and moment of inertia about orthogonal axes aligned with the pelvic coordinate system relevant to the segment’s centre of mass) were estimated by a MATLAB script based on the divergence theorem, which converts surface integrals into equivalent volume integrals (Fig. 2, workflow B) (Semechko 2025). Estimated body segment inertial parameters (Appendix A2 in Supplementary Material) were subsequently imported into the corresponding OpenSim pelvic segments. The inertial properties of the sacrum were calculated based on the pelvis density together with the average sacrum volume but not subject-specifically scaled, which may introduce minor errors in estimating the joint reaction forces.

### Opensim simulation

After updating the pelvis segment inertial parameters of the OpenSim model, joint kinematics were computed using the Inverse Kinematics tool. The Residual Reduction Algorithm was then used to generate a smoothed set of kinematics that reduced dynamic inconsistency between the measured kinematic and kinetic data by adjusting the inertial properties of the torso segment (Delp et al. 2007). Muscle forces were estimated using the Static Optimization tool (Delp et al. 2007). Lastly, the Joint Reaction Analysis tool was used to estimate the sacroiliac and pubic joint compression and shear forces (Delp et al. 2007). The SIJ compressive/tensile force was defined along the Z-axis (the normal vector of the best-fitted plane of the articular surface), and the SIJ shear force was defined within the X–Y plane (best-fitted plane of the articular surface) (Fig. 3A right view). Notably, for the right SIJ, compressive forces were defined as acting towards the sacrum (− Z), and tensile forces as acting away from the sacrum (+ Z); the opposite convention was applied for the left SIJ due to the right-hand rule used in OpenSim joint definitions. The shear force component along the X-axis represented the anterior–posterior shear, and the component along the Y-axis represented the superior–inferior shear (Fig. 3A). All joint forces were expressed in their parent body coordinate system (Fig. 3A&B).

Simulation outcomes reported in this study included compressive/tensile and superior–inferior shear reaction forces acting on the sacroiliac and pubic joints during bilateral standing, single-leg standing, and walking. For the static tasks (bilateral and single-leg standing), a stable 3-s interval without substantial movement fluctuations was selected from each trial for simulation. For each participant, results for each static task were averaged across two repeated trials. For walking, one complete right gait cycle was selected per trial. A right gait cycle was defined from the initial contact (by applying a 20 N threshold to the vertical component of the ground reaction force (Vrahas et al. 1995)) of the right foot to the subsequent initial contact of the same foot. For each participant, three gait cycles were averaged. The joint reaction forces of each subject were normalised by their total body mass (N/kg).

### Model evaluation

The geometric accuracy of the SSM-based pelvic scaling workflow was evaluated using a leave-one-out cross-validation analysis using CT-derived pelvis meshes as ground truth, and compared with conventional linear scaling approaches (details in Appendix B in Supplementary Material). The primary comparison was performed between the SSM-based approach and a linearly scaled female pelvis to evaluate the additional contribution of shape-based personalisation beyond conventional linear scaling. The linear scaling of a generic male OpenSim pelvis was included as an additional reference to evaluate the influence of sex-specific pelvic morphology (Appendix B in Supplementary Material). Reconstruction accuracy was quantified using pelvis surface root mean square error (RMSE) and three-dimensional Euclidean distance errors of pelvis-related joint centres (SIJs, pubic symphysis, L5–S1, and hip joint centres). Differences between the SSM-based and linearly scaled female pelvis were assessed using one-tailed paired t-tests.

The capability of the model to predict pelvic joint loading was further evaluated by comparing the simulated pelvic joint reaction forces with values reported in previous in vitro and computational modelling studies during bilateral standing, single-leg standing, and walking. The results used for comparison were expressed in the ‘evaluating’ joint coordinate system (Fig. 3A-1), aligned with previous studies (Scholten et al. 1988; Meissner et al. 1996). Furthermore, pelvic joint reaction forces were normalised by the trunk mass (M_trunk_) or total body mass (M_total body_), depending on the normalisation convention used in the reference studies. The mean trunk mass of the participants in the present study was 33.4 ± 8.2kg obtained from the scaled models.

For symmetric bilateral standing, the mean value of the left and right SIJ reaction forces was used for comparison with the previous literature. In single-leg standing, only the supporting side was considered. For static tasks (bilateral or single-leg standing), the comparisons focused on the mean joint reaction forces over a 3-s time period. For walking, the comparison was based on the absolute mean of the peak reaction force across gait cycles.

## Results

To facilitate comparison with previous studies, pelvic joint reaction forces reported in Sect. 3.1 were expressed in the ‘evaluating’ joint coordinate system and normalised according to conventions used in the literature (i.e. by the trunk mass or total body mass).

In contrast, the results presented in Sects. 3.2–3.4 are reported in the ‘anatomically aligned’ coordinate system and normalised by total body mass, to enable consistent comparisons across different conditions.

### Model evaluation

#### Pelvis geometry reconstruction

The SSM-based approach achieved a mean surface reconstruction error of 6.88 ± 2.44 mm, compared with 7.13 ± 2.36 mm for the linearly scaled female pelvis (Table 1). The improvement in surface RMSE was therefore modest (0.25 mm, p = 0.03).

**Table 1:** Pelvis reconstruction and joint centre errors for SSM-based and linear scaling methods

	RMSE (mm)	SIJ_l (mm)	SIJ_r (mm)	PS (mm)	L5-S1 (mm)	HJC_l (mm)	HJC_r (mm)
SSM-based scaling	6.88 ± 2.44	12.15 ± 6.01	12.39 ± 6.36	14.58 ± 6.83	13.58 ± 6.2	12.97 ± 6.19	13.32 ± 5.87
Linear female pelvis scaling	7.13 ± 2.36	12.3 ± 6.2	12.72 ± 6.44	15.68 ± 7.26	13.78 ± 6.12	14.3 ± 6.35	14.82 ± 6.08
p-value	0.03	0.38	0.09	0.02	0.04	< 0.01	< 0.01

Values are presented as mean ± standard deviation. Pelvis surface error was quantified using root mean square error (RMSE). Joint centre errors were computed as three-dimensional Euclidean distance (SIJ: sacroiliac joint; PS: pubic symphysis; HJC: hip joint centre). *p-values* were obtained from one-tailed paired t-tests comparing each linear scaling method against the SSM-based scaling approach. p < 0.05 was considered statistically significant. Comparisons with the linear-scaled generic male OpenSim pelvis are provided in Appendix B (Table B1 in Supplementary Material)

For pelvis-related joint locations, the SSM-based approach showed comparable SIJ centre errors to the linearly scaled female pelvis (p > 0.05). Small but statistically significant improvements were observed for the PS, L5–S1 joint, and hip joint centres, with the largest improvement observed for the hip joint centres, where errors were reduced by approximately 1.3–1.5 mm compared with female linear scaling (both p < 0.01).

#### During bilateral standing

During bilateral standing, a previous biomechanical model reported a SIJ compressive force of 0.87 N/kg (Scholten et al. 1988), our model predicted a similar value of 0.7 N/kg (Table 2). Regarding SIJ shearing, the FE model reported anterior–posterior and superior–inferior shear forces of 0.47 N/kg and 4.77 N/kg, respectively (Scholten et al. 1988), while our model estimated 0.23 N/kg and 7.81 N/kg for these components (Table 1).

**Table 2:** Comparison of pelvic joint reaction forces during different activities across references

	Joint /Joint force type	Normalisation	Joint forces from the present study	Joint forces from the literature
Bilateral standing	SIJ (both sides) /compressive	/M_trunk_	0.70 ± 0.59 N/kg	0.87 N/kg (Scholten et al. 1988)
	SIJ (both sides) /anterior–posterior shear	/M_trunk_	0.23 ± 0.54 N/kg	0.47 N/kg (Scholten et al. 1988)
	SIJ (both sides) /superior–inferior shear	/M_trunk_	7.81 ± 1.13 N/kg	4.77 N/kg (Scholten et al. 1988)
Single-leg standing	SIJ (support side) /resultant	/M_total body_	5.03 ± 1.48 N/kg	8.33–10.79 N/kg (Goel and Svensson 1977)
	PS/compressive	/M_total body_	2.24 ± 0.39 N/kg	0.59–2.48 N/kg (Goel and Svensson 1977)
	PS/superior–inferior shear	Absolute value	87 ± 141 N	148 N (Meissner et al. 1996)
	PS/anterior–posterior shear	Absolute value	84 ± 9 N	169 N (Meissner et al. 1996)
Walking	PS/peak superior–inferior shear	Absolute value	170 ± 73 N	136 N (Meissner et al. 1996)
	PS/peak anterior–posterior shear	Absolute value	35 ± 33 N	333 N (Meissner et al. 1996)

SIJ: sacroiliac joint; PS: pubic symphysis; F_L5-S1_: participant-specific vertical force transmitted through the L5-S1 joint; M_total body_: total body mass. All the forces were expressed in the pelvic ‘evaluating’ joint coordinate system (Fig. 3A-1)

At this stage, the mean medial–lateral hip joint reaction force, averaged across the left and right sides, was 2.14 ± 0.78 N/kg, with both sides demonstrating medially directed (compressive) forces.

#### During single-leg standing

A previous pelvis modelling study reported SIJ resultant forces ranging from 8.33 to 10.79 N/kg during single-leg standing (Goel and Svensson 1977), while our prediction of 5.03 N/kg was lower (Table 2).

For the pubic joint, compressive forces have been reported between 0.59 and 2.48 N/kg (Goel and Svensson 1977), with the present estimate of 2.24 N/kg approaching the upper limit of this range. In vitro experiments further indicated that 169 N superior–inferior and 148 N anterior–posterior shear forces were required to induce mean pubic joint mobility during single-leg standing (Meissner et al. 1996), while our estimated pubic shearing (87 N superior–inferior, 84 N anterior–posterior) was relatively lower.

#### During walking

Previous in vitro experiments reported that superior–inferior and anterior–posterior forces of 333 N and 136 N, respectively, were required to induce maximal pubic mobility during walking (Meissner et al. 1996). In the present study, the peak pubic joint superior–inferior and anterior–posterior shear forces during walking were 35 N and 170 N, respectively (Table 2).

### Pelvic joint reaction forces during bilateral standing

**Fig. 4:**
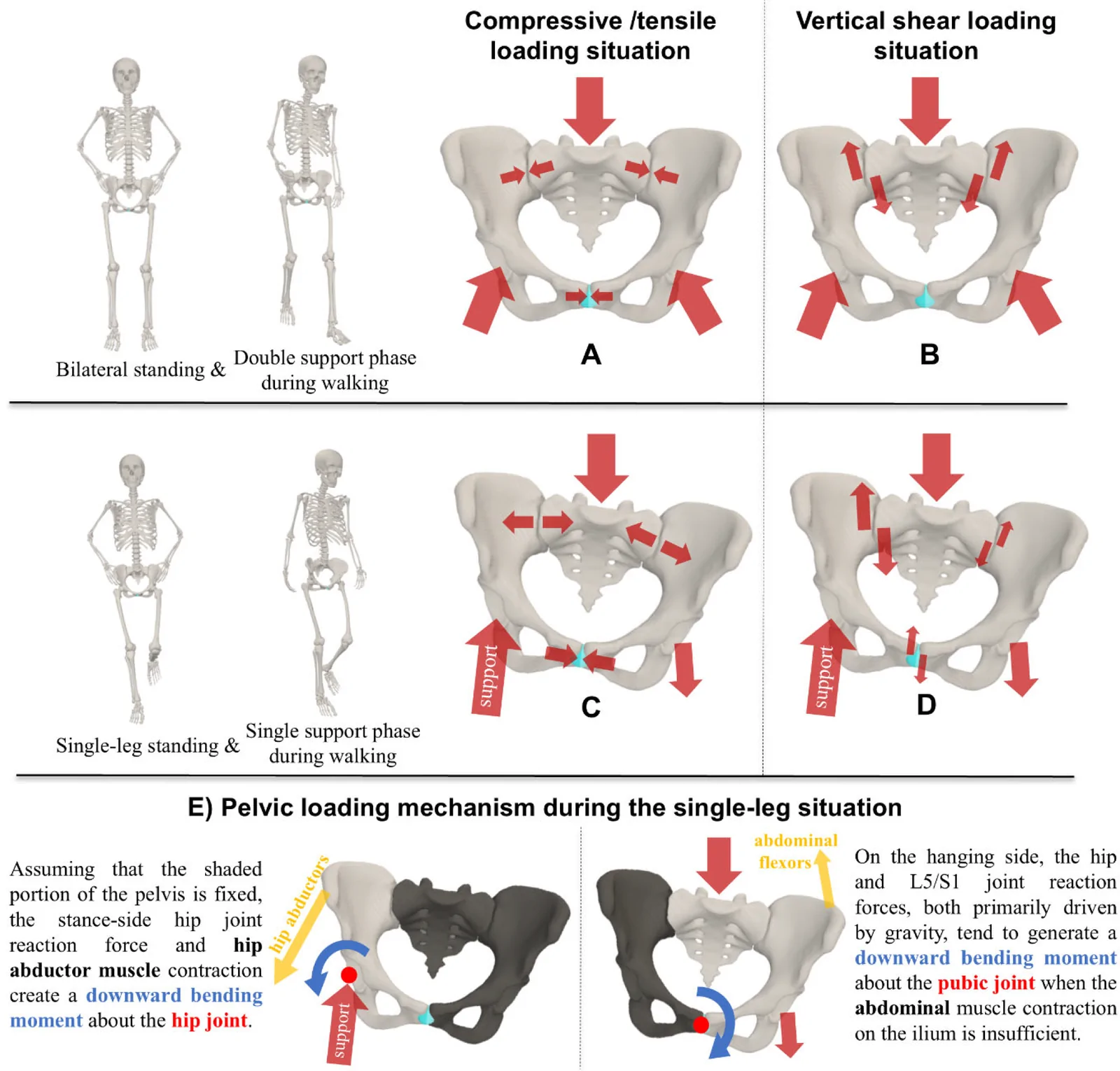
Diagram of pelvic joint loading situation of the pelvic model in the coronal plane during different activities (A, B, C, D), and (E) the potential pelvic loading mechanism during the single-leg situation. A) and B) are the pelvic joint compressive and superior–inferior shear loading situations during bilateral standing or double-support phase of walking; C) and D) are the pelvic joint compressive and superior–inferior shear loading situations during single-leg standing or single-support phase of walking. The pelvic orientation illustrated (inclination towards the hanging side) represents the predominant posture during single-leg situations (mean inclination: 1.1 ± 1.5° during single-leg standing; 2.3 ± 0.9° during the single-support walking phase); however, three participants exhibited pelvic inclination towards the support side during single-leg standing (mean inclination: 3.8 ± 1.9°)

During bilateral standing, the pelvic model predicted low SIJ compressive/tensile forces on both sides, each below 0.3 N/kg (Table 3, Fig. 4). Although the mean forces on both sides indicated compressive forces, high inter-subject variability remained. The superior–inferior shear forces at the SIJs were also similar between sides, averaging 3.35 N/kg and directed upward from each hemipelvis towards the sacrum (Table 3, Fig. 4).

**Table 3:** Normalised sacroiliac and pubic joint reaction forces during static bilateral and single-leg standing

	Bilateral standing	Single-leg standing (right-leg support)
SIJ_r superior–inferior shear force	3.21 ± 0.48 N/kg	4.01 ± 0.65 N/kg
SIJ_r compressive/tensile force	-0.05 ± 0.28 N/kg (compressive)	1.85 ± 0.34 N/kg (tensile)
SIJ_l superior–inferior shear force	3.49 ± 0.51 N/kg	2.43 ± 0.64 N/kg
SIJ_l compressive/tensile force	0.28 ± 0.30 N/kg (compressive)	-1.96 ± 0.34 N/kg (tensile)
Pubic joint superior–inferior shear force	0.02 ± 0.37 N/kg	0.64 ± 1.82 N/kg
Pubic joint compressive/tensile force	0.34 ± 0.41 N/kg (compressive)	2.24 ± 0.39 N/kg (compressive)

SIJ_r: right sacroiliac joint; SIJ_l: left sacroiliac joint; All the forces were expressed in the pelvic ‘anatomically aligned’ joint coordinate system (Fig. 3A-2)

### Pelvic joint reaction forces during single-leg standing

During single-leg standing, the pelvic model predicted similar SIJ tensile forces between the supporting and hanging sides, averaging 1.91 N/kg (Table 3, Fig. 4). Superior–inferior shear forces were much higher on the supporting side (4.01 N/kg) than on the hanging side (2.43 N/kg), all directed upward from each hemipelvis to the sacrum (Table 3, Fig. 4).

At the pubic joint, superior–inferior shear forces averaged 0.64 ± 1.82 N/kg, while compressive forces averaged 2.24 ± 0.39 N/kg (Table 3, Fig. 4). Although the mean direction of the superior–inferior shear forces across subjects was directed upward from the right hemipelvis to the left, the large variability indicates substantial inter-subject differences in both magnitude and direction.

### Pelvic joint reaction forces during walking

**Fig. 5:**
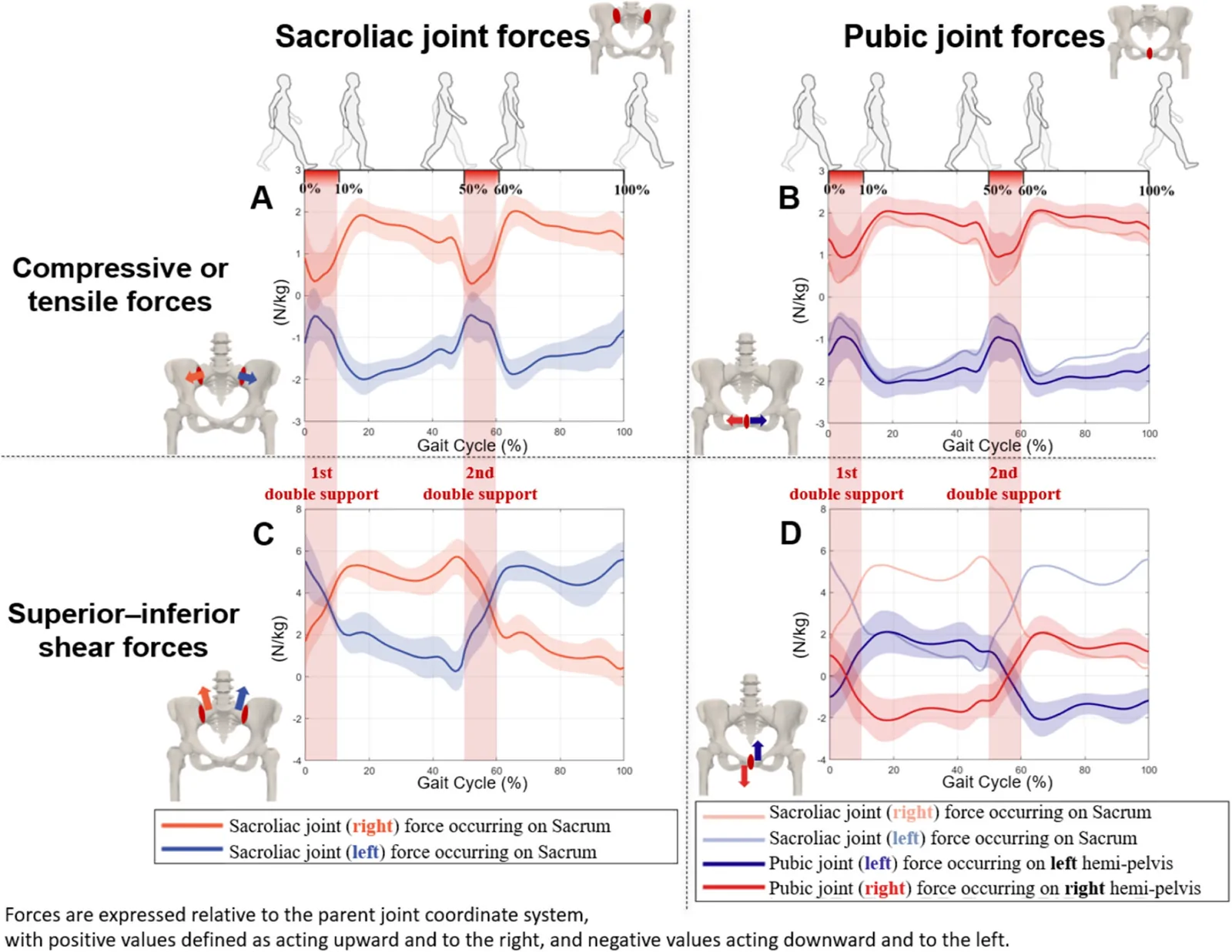
Pelvic joint reaction forces (N/kg) during one right gait cycle. A) and B) are the compressive/tensile forces of the SIJ and pubic joint, respectively; C) and D) are the superior–inferior shear forces of the SIJ and pubic joint, respectively. All the forces were normalised to the total body mass (N/kg), and expressed in the pelvic ‘anatomically aligned’ joint coordinate system (Fig. 3A-2)

During walking, the right SIJ exhibited tensile force, oriented away from the sacrum along the positive SIJ Z-axis (+ Z). The magnitude first decreased to near zero (0.5 N/kg) during the first double-support phase, after which it increased during the transition from double support to single support. The force subsequently remained at a relatively high level throughout the ensuing single-support phase with a peak of 2 N/kg (Fig. 5A). A similar cyclic pattern was observed during the remaining half of the gait cycle, characterised by a reduction of the tensile force during the double-support phase and subsequent increase during the swing phase, followed by a sustained high magnitude. The left SIJ exhibited an analogous temporal pattern, with tensile forces similarly oriented away from the sacrum but along the negative SIJ Z-axis (-Z).

For the pubic joint, the force was predominantly compressive, with a temporal pattern comparable to the SIJ tensile forces, but with slightly higher magnitudes, reaching a peak of 2.1 N/kg (Fig. 5).

The right SIJ superior–inferior shear force was directed upward (+ Y) relative to the sacrum. The magnitude gradually increased after heel strike, reaching a peak of 5.7 N/kg at the end of the right stance phase, and then decreased during the subsequent swing phase, reaching a minimum of 0.3 N/kg at the end of the right gait cycle (Fig. 5C). The left SIJ superior–inferior shear forces exhibited the same upward (+ Y) direction relative to the sacrum and followed a temporal pattern that mirrored that of the right SIJ, but occurred during the contralateral (left) gait cycle.

For the pubic joint superior–inferior shear forces (from the left to the right hemipelvis), an initial upward (+ Y) shearing switched to a downward (-Y) direction during the first double support (from left foot contact to right toe-off), reaching a negative peak of -2.1 N/kg after double support (right toe-off) (Fig. 5). The force then remained at a relatively high level until the next double support (from right foot contact to left toe-off), where it dropped sharply and reversed direction, attaining a positive peak of 2.1 N/kg after this double support (right toe-off) (Fig. 5).

## Discussion

This study developed and evaluated a female pelvis musculoskeletal model based on the ‘pelvic ring’ theory to investigate the reaction forces at the sacroiliac joint (SIJ) and pubic symphysis (PS) during bilateral standing, single-leg standing, and walking. The model produced broadly plausible estimates of sacroiliac and pubic joint reaction forces —primarily compressive/tensile and superior–inferior shear forces— when compared with previous in vitro, in vivo, FE and musculoskeletal modelling results. Key observations included: (1) During bilateral standing, pubic joint reaction forces were low, and SIJ reaction forces were relatively balanced between sides; (2) during single-leg standing, the SIJs experienced tensile loading, whereas the pubic joint experienced compression, accompanied by high stance-side SIJ shear forces and the pubic shear forces with high variability; and (3) during walking, pubic joint reaction forces were closely related to SIJ forces, transmitting superior–inferior shearing from the stance-side SIJ to the swing-side SIJ.

### Model evaluation

#### Geometry reconstruction evaluation

The SSM-based approach and the linearly scaled female pelvis performed comparably in reconstructing pelvic geometry and joint centres using four superior iliac landmarks. Although statistically significant improvements over linear female scaling were observed for several joint centre localisations, these improvements were generally modest relative to the overall joint centre errors (approximately 12–15 mm) observed for both approaches. Both approaches substantially improved geometric reconstruction compared with the generic male OpenSim pelvis (Appendix B in Supplementary Material). Nevertheless, residual joint centre localisation errors may still introduce uncertainty in the estimation of corresponding muscle moment arms and, consequently, in joint reaction force estimates.

#### Joint loading evaluation

The plausibility of the proposed pelvic model was evaluated by comparing the predicted joint reaction forces (expressed in the ‘evaluating’ joint coordinate system, Fig. 3A-1) with previously reported modelling or in vitro results (Table 2).

During bilateral standing, the predicted SIJ compressive forces were comparable to values reported in previous biomechanical modelling studies (Scholten et al. 1988). The present model predicted relatively higher superior–inferior shear forces than previous models. This discrepancy may be attributable to differences in loading conditions, as previous studies applied external loads primarily along the longitudinal axis of the sacrum and did not account for muscle forces, which does not fully replicate physiological standing. Nevertheless, both the present and previous models identified superior–inferior shear as the dominant SIJ loading component, supporting the plausibility of the predicted loading pattern under symmetric standing conditions.

During asymmetric single-leg standing, when compared with the previously published mathematical model (Goel and Svensson 1977), the present model predicted higher pubic joint compressive forces and lower SIJ resultant forces. These discrepancies may partly reflect methodological differences between the models. The previous model estimated SIJ resultant force from ground reaction forces using a unilateral lower-limb representation (Goel and Svensson 1977). Compared with the present model, it assumed a lower proportion of lower-body mass (40% of total body mass versus 52.7% in the present model), which may have contributed to higher estimated SIJ loading. Furthermore, the contralateral limb was not explicitly represented, and the pubic symphysis was modelled as an element capable of transmitting only axial tensile/compressive forces. These assumptions limited load transfer from the support side to the contralateral hemipelvis (no contralateral hemipelvis in this model) through the pubic symphysis and likely shifted a greater proportion of the load to the support-side SIJ. When compared with the available in vitro measurements (Meissner et al. 1996), the predicted pubic superior–inferior shear force was slightly lower. However, the large standard deviation observed in the present cohort suggests substantial inter-subject variability, likely reflecting differences in postural strategies (e.g. pelvic inclination and non-supporting limb position) during single-leg standing, as further discussed in Sect. 4.3.

During walking, estimated superior–inferior peak pubic joint forces were comparable to the in vitro values (Meissner et al. 1996). However, anterior–posterior shear forces were significantly lower than previously reported values. Similar underestimation was also observed during bilateral standing and single-leg standing, indicating a systematic discrepancy in the prediction of anterior–posterior shear loading. This pattern partly reflects limitations of the present pelvic joint functional modelling approach in representing load transfer in the anterior–posterior direction (see 4.5 Limitations). Meanwhile, for bilateral standing/walking, the standard deviation of pubic anterior–posterior shear exceed/approaches its mean, indicating substantial variability in both magnitude and direction. This may be attributed to the high sensitivity of anterior–posterior shear to coordinate system definition, where differences between the evaluating and anatomically aligned coordinate systems can reach up to 244% (Appendix C in Supplementary Material), as well as reconstruction-related uncertainties in pelvic coordinate definition (Appendix B in Supplementary Material). Collectively, these findings indicate that anterior–posterior shear is less robust than other loading components, although this loading type is not the primary focus of the present study.

Overall, the estimated joint reaction forces reproduced the general loading patterns reported in previous studies. Agreement was better for compressive/tensile and superior–inferior shear loading, whereas anterior–posterior shear exhibited systematic underestimation and higher sensitivity to modelling and coordinate system definitions. These results suggest the plausibility of the modelling approach for the primary loading measures examined in this study, particularly compressive/tensile and superior–inferior shear loading.

### Pelvic joint reaction forces during bilateral standing

During bilateral standing, in terms of mean forces, the pelvic model predicted low SIJ compressive forces (less than 0.3 N/kg) accompanied by higher superior–inferior shear forces (higher than 3 N/kg) on both sides, which were consistent with those reported in the literature (Scholten et al. 1988; Pel et al. 2008a). Although the mean SIJ axial loading indicated compression, considerable inter-subject variability was observed. Given the relatively small magnitude of the compressive/tensile component, it may be more sensitive to asymmetric standing postures in a limited number of participants.

Notably, a wide variation in SIJ superior–inferior shear forces has been observed across the literature (Scholten et al. 1988; Pel et al. 2008a). Such discrepancies may arise from differences in model configurations or joint coordinate system definitions, but are also likely attributable to variations in how muscle activation is set across models. For instance, one modelling study employed different muscle optimisation strategies and demonstrated that the activation pattern of trunk and pelvic muscles substantially influences SIJ loading (Pel et al. 2008a).

At the pubic joint, the model predicted low compressive/tensile loading (0.34 ± 0.41 N/kg) and negligible superior–inferior shear (0.02 ± 0.37 N/kg), both accompanied by substantial inter-subject variability. In particular, the standard deviation of pubic compressive/tensile loading exceeded the mean value, indicating that both compressive and tensile loading patterns were present across participants. Similar disagreement exists in the literature. While Ulmer et al. (Ulmer et al. 1993) reported compressive loading across the pubic symphysis, several in vitro studies have reported tensile stresses during bilateral standing (Icke and Koebke 2014; Varga et al. 1995).

These differences may partly reflect variations in loading conditions. Cadaveric experiments typically employ simplified boundary conditions, such as applying superior–inferior loads through the acetabula (Ulmer et al. 1993) or constraining the distal pelvis while applying a downward force to the sacrum (Icke and Koebke 2014). In contrast, the present full-body musculoskeletal model predicted hip joint reaction forces containing both superior–inferior and medially directed components (medial hip joint force = 2.14 ± 0.78 N/kg). This inward-directed loading may contribute to compressive loading across the pubic symphysis in some participants and may partly explain why compression was observed on average in the present cohort.

The negligible mean pubic superior–inferior shear force with relatively large standard deviation is expected, as bilateral standing is a symmetric posture with a theoretical minimal left–right differences in loading, and this imbalance varies across different subjects.

### Pelvic joint reaction forces during single-leg standing

During single-leg standing, the model predicted similar tensile forces at both SIJs, accompanied by higher superior–inferior shearing on the support side and lower shearing on the contralateral side. One FE model integrating muscle forces and joint reaction forces derived from inverse kinetics similarly estimated that the stance-side SIJ was primarily subjected to superior–inferior shear and tensile forces, consistent with the present simulation (Ricci et al. 2020). In contrast, the hanging-side SIJ in that model experienced compressive forces (Ricci et al. 2020), which may be attributed to high activation of trunk muscles on the hanging side (Fig. 4E). Several in vitro experimental studies have documented widening of the stance-side SIJ under simulated single-leg standing conditions (MacAvoy et al. 1997; Yinger et al. 2003). Although additional joint fixation or partial removal of soft tissues was applied in these cadaver experiments (MacAvoy et al. 1997; Yinger et al. 2003), the results indicate that SIJ tensile forces during single-leg standing are physiologically plausible.

For the pubic joint, compressive force represented the most consistent loading component, whereas superior–inferior shear showed substantial inter-subject variability. The mean loading pattern aligns with many in vitro observations and FE modelling, which similarly identify compression and superior–inferior shear as the primary loading modes at the PS (Ricci et al. 2020; Icke and Koebke 2014; Fabeck et al. 1994; MacAvoy et al. 1997; Vrahas et al. 1995; Goel et al. 1978; Jordan et al. 2021). Observations from in vivo imaging further support this interpretation, as a caudal displacement of the PS has been observed at the hanging leg during single-leg standing (Mens et al. 1999). Synthetic pelvis models have also demonstrated gap closure under simulated single-limb loading (Godinsky et al. 2018). Similarly, an early musculoskeletal model that allowed the PS to transmit only tension and compression reported compressive loading throughout single-leg stance (Goel and Svensson 1977). In the present simulation, compressive loading was greater than shear at the PS (Table 3), consistent with FE findings (Ricci et al. 2020).

However, substantial inter-subject variability was observed in pubic superior–inferior shear loading. This variability likely reflects differences in the postural strategies participants adopted during single-leg standing. Specifically, participants demonstrated varying degrees of pelvic inclination (captions in Fig. 4) and different strategies for positioning the non-supporting limb. For instance, some participants maintained the swing limb by hip flexion, whereas others adopted a knee-flexed posture with hip extension. These postural variations likely alter muscle recruitment patterns and load transfer pathways across the pelvic ring, thereby contributing to the observed variability in pubic shear loading.

Due to the asymmetry, single-leg standing generates considerable bending moments across the pelvis (Ries et al. 1989; Goel et al. 1978; McLeish and Charnley 1970). The downward bending moment in the stance-side ilium, arising from abductor muscle contraction and the associated joint reaction force (Ries et al. 1989), together with the gravitational effect of the hanging-side hemipelvis, produces an external downward bending moment about the pubic joint in the coronal plane (Fig. 4E). This mechanism may contribute to the mean pattern of tensile forces at the SIJs, the compressive forces at the pubic joint, and the superior–inferior shear forces at both joints during single-leg standing (Fig. 4C&D).

However, this situation (tensile forces at both SIJs) may not be the only scenario for SIJ axial compressive/tensile loads during single-leg standing. Indeed, previous studies have demonstrated that variations in trunk muscle activation strategies can remarkably influence pelvic joint loading (Pel et al. 2008a). During single-leg standing, contraction of the lumbar and abdominal muscles on the hanging-side hemipelvis can generate an internal bending moment that counteracts or even surpasses the external bending moment induced by gravity (Fig. 4E). This mechanism potentially explains why some models predict compressive rather than tensile loading at the hanging-side SIJ (Ricci et al. 2020). More broadly, inter-individual differences in trunk muscle recruitment and postural control strategies may also contribute to the substantial variability in pubic superior–inferior shear loading observed in the present cohort.

### Pelvic joint reaction forces during walking

The gait cycle can be interpreted as a continuous alternation between single-support and double-support phases (Fig. 5). During walking, the temporal pattern in pelvic joint reaction force from single support to double support, particularly in the coronal plane, is broadly comparable to the transition from single-leg to bilateral standing (Fig. 4).

For superior–inferior shear forces, both sides of the SIJ forces were directed upward relative to the sacrum. During single support, the stance-side SIJ shearing remained high, while the contralateral SIJ shearing remained low, and load distribution between the two sides switched during double support. Pubic joint superior–inferior shearing was relatively high during single support, acting upward from the stance-side pelvis towards the contralateral side, but underwent a sharp directional reversal during the double-support phase.

Regarding the compressive/tensile components, both SIJs experienced tension, with higher values during single support and lower values during double support. At the same time, the pubic joint exhibited compression, following a similar changing pattern, with elevated values in single support and reduced values in the double-support phase. Although not a direct measure of SIJ reaction forces, one FE model (Toyohara et al. 2020) demonstrated a separation tendency of the SIJ cartilage in both the stance and swing phases, with separating deformation in the lower region during stance and separating deformation in the upper region during swing.

Pubic joint forces were strongly correlated with SIJ forces. For compressive/tensile forces, variations in pubic joint compression closely mirrored the tensile forces at both SIJs, reflecting the corresponding downward bending moment of the swing-side hemipelvis about the pubic joint during single support (Fig. 4E). For superior–inferior shear forces, pubic joint values closely matched those of the swing-side SIJ, suggesting that during single-leg support, the pubic joint may help transmit superior–inferior shearing from the high-loading stance-side SIJ to the swing-side SIJ.

This potential load transmission role of the PS is supported by in vitro experiments evidence (MacAvoy et al. 1997), where an intact or fixed symphysis preserved stance-side SIJ stability during single-leg standing, while disrupted symphyses led to immediate failure of the stance-side SIJ ligaments, likely because the pubic joint transmitted the high SIJ superior–inferior shear from the stance side to the opposite side. Another study looking at FE model simulation further supports this mechanism: during walking, the superior and inferior pubic rami play an essential role in load transmission through the PS in a healthy pelvis, but not in pelvises with ramus fractures (Ricci et al. 2018).

Given the circular ring structure of the pelvis, it is unsurprising that a loading change at one point induces effects at other locations. The results from the present simulation, especially the asymmetrical movement (single-leg standing and walking), further support the findings from Ricci et al. that there is a close biomechanical interdependence between both SIJs and the PS (Ricci et al. 2018, 2020).

### Limitations

The ‘pelvic ring’ is a closed-loop structure constrained by numerous strong ligaments, interlocking joint surfaces, and the mutual constraint of the three pelvic joints. In the present model, the constraint of the ‘pelvic ring’ was represented only by the mutual constraints among the three pelvic joints within the plane of the ‘pelvic ring’ (the X–Z plane in joint functional coordinate systems, Fig. 3B), while neglecting the contributions of the ligaments and articular surface morphology. Within this rigid closed-loop formulation, the distribution of joint reaction forces between the sacroiliac and pubic joints is governed by the chosen constraint topology rather than tissue-level mechanical properties. This simplification likely overestimates the capacity of the three joints to transmit loads within this ‘pelvic ring’ plane.

Beyond passive structures, contraction of the pelvic floor muscles can increase pelvic ring stiffness and stability (Pool-Goudzwaard et al. 2004), and their co-contraction with abdominal muscles can modulate intra-abdominal pressure (Easley et al. 2017), thereby influencing pelvic joint loading (Pel et al. 2008a; Pool-Goudzwaard et al. 2004). However, in the present model, the entire pelvis was constrained as a rigid body without SIJ motion, so pelvic floor muscle forces could not be meaningfully computed by static optimisation. In addition, a more accurate representation for intra-abdominal pressure modelling is beyond the current scope of the OpenSim software (Christophy et al. 2012). Therefore, this study did not include pelvic floor muscles, or intra-abdominal pressure, which may influence pelvic joint loads (Eichenseer et al. 1976; Pel et al. 2008a; Pool-Goudzwaard et al. 2004; Easley et al. 2017).

The pelvis-related muscle paths were not fully subject-specific, as muscle origin locations were transferred from a generic male-based model and muscle via points were repositioned using a linear scaling approach. Consequently, female- and subject-specific variations in muscle geometry and musculotendon architecture were not fully represented, which may have influenced the estimation of muscle forces and pelvic joint reaction forces. In addition, the static optimisation neglected muscle co-activation, particularly underestimating the activation of the core (‘lumbopelvic–hip complex’) (Kibler et al. 2006), which may further influence pelvic joint loading (Pool-Goudzwaard et al. 2004). For instance, during single-leg standing, insufficient core muscle activation may allow gravity to generate a high downward bending moment on the hanging-side hemipelvis (Fig. 4E), potentially causing the model to overestimate SIJ tensile forces, pubic joint compressive forces, and shear forces across the pelvis. Future studies could improve muscle force estimation by incorporating subject-specific muscle geometry and more physiologically realistic neuromuscular control strategies beyond static optimisation.

In the present study, a uniform 5 mm soft tissue correction was applied to both the ASIS and PSIS landmarks during pelvic reconstruction. However, soft tissue thickness and landmark palpation uncertainty may vary across pelvic landmarks and individuals, particularly in female populations. Because the reconstruction workflow relied on these four pelvic landmarks, inaccuracies in soft tissue correction may introduce systematic errors in estimated pelvic geometry, which could subsequently influence the definition of pelvic joint coordinate systems and predicted joint loads.

It should be noted that sacral mass and inertia were estimated using a uniform density assumption and an average sacral volume derived from the SSM training dataset, rather than being obtained through subject-specific segmentation. This introduces a degree of modelling uncertainty in the absolute magnitude of predicted joint reaction forces. However, as the sacrum primarily functions as a load-transmitting segment within the pelvic ring and its mass is relatively small compared with the hemipelves, the influence of this assumption on comparative loading trends across conditions is expected to be limited. Future sensitivity analyses investigating the effect of sacral inertial properties on pelvic joint loading would be valuable.

Another limitation of the present study is the small sample size and the limited standardisation of certain experimental tasks. In particular, participants were not required to adopt a specific postural strategy during single-leg standing, resulting in variations in pelvic inclination and non-supporting limb position. These differences likely contributed to the relatively large inter-subject variability observed in some joint reaction force components. Therefore, some biomechanical findings were interpreted primarily as overall loading patterns and characteristic trends rather than precise representations of a single underlying mechanical mechanism. Future studies with larger sample sizes and more strictly standardised movement protocols are warranted to further investigate the mechanisms underlying pelvic load transfer.

A formal sensitivity analysis was not performed in this study but would be valuable for quantifying the influence of modelling assumptions. Future studies should systematically evaluate the sensitivity of pelvic loading estimates to key model parameters, including joint definitions and inertial properties, to better assess the robustness of model predictions.

## Conclusions

This study developed and evaluated a female pelvis musculoskeletal model with sacroiliac and pubic joints, integrated with an SSM-based workflow for personalised shape scaling and an inertia estimation workflow using 3D full-body scans. The model generated broadly plausible predictions of pelvic joint reaction forces, particularly compressive and superior–inferior shear loads, during bilateral standing, single-leg standing, and walking. Pubic joint reaction forces were minimal during bilateral standing but became substantial during asymmetrical single-leg standing, particularly in superior–inferior shear and compression. In contrast, SIJ forces during single-leg standing were dominated by tensile and superior–inferior shear forces. During walking, pubic joint forces were strongly correlated with SIJ forces, suggesting that the pubic joint may contribute to transmitting loading from the stance-side SIJ to the swing-side SIJ.

Overall, the proposed modelling framework provides a feasible tool for investigating pelvic joint reaction forces in women and can be adapted to other populations, offering a foundation for future studies on pelvic pain mechanisms, pregnancy-related adaptations, and sex-specific musculoskeletal disorders.

## Supplementary Information

Below is the link to the electronic supplementary material.Supplementary file1 (DOCX 933 KB)
