# Supplementary material for: Development of a pelvic musculoskeletal model based on statistical shape modelling for estimating sacroiliac and pubic joint reaction forces in females
Source: Biomech Model Mechanobiol. 2026 Aug 5;25(4):91. doi: 10.1007/s10237-026-02110-5 (PMC13442515; doi:10.1007/s10237-026-02110-5)
Supplement: Supplementary file 1 [file 10237_2026_2110_MOESM1_ESM.docx]

## Appendix A

### 1. Manually delineated joint contact regions

The acetabular, auricular, symphyseal, and L5-S1 regions were manually delineated on the mean pelvis mesh using identifiable articular surface boundaries. Although manual delineation may introduce some operator-dependent variability, the joint definitions were derived from geometric features fitted to the entire selected surface regions (e.g., best-fit spheres and planes), which reduces sensitivity to local selection inaccuracies. Consequently, small variations in the delineated boundaries are unlikely to substantially affect the resulting joint definitions.

Figure A1. Visualization of manually delineated L5-S1, acetabular, auricular, and symphyseal joint regions in the mean pelvis mesh

### 2. Segmental inertial properties of the pelvis estimated from 3D scan

Table A2. Segmental inertial properties of the sacrum and hemi-pelvis expressed in the pelvis coordinate system (n = 8, mean ± SD)

| Segment | Mass  (kg) | CoM x/y/z  (m) | Ixx  (kg·m² ×10⁻³) | Iyy  (kg·m² ×10⁻³) | Izz  (kg·m² ×10⁻³) | Ixy  (kg·m² ×10⁻³) | Ixz  (kg·m² ×10⁻³) | Iyz  (kg·m² ×10⁻³) |
| --- | --- | --- | --- | --- | --- | --- | --- | --- |
| Sacrum | 0.194 | -0.108 /0 /0 | 0.267 | 0.216 | 0.174 | -0.061 | 0.002 | 0 |
| Left hemipelvis | 3.547 ± 0.346 | -0.059 ± 0.010 /-0.046 ± 0.003 /-0.056 ± 0.004 | 14.402 ± 1.81 | 25.166 ± 5.266 | 27.713 ± 5.866 | -2.429 ± 1.143 | -0.016 ± 0.565 | 3.001 ± 0.417 |
| Right hemipelvis | 3.791 ± 0.586 | -0.057 ± 0.009 /-0.046 ± 0.006 /0.055 ± 0.003 | 16.220 ± 3.457 | 26.978 ± 7.495 | 30.360 ± 8.435 | -2.826 ± 1.220 | 0.466 ± 0.548 | -3.189 ± 0.884 |

## Appendix B

### 1. Validation procedure of the pelvis geometry reconstruction

To quantify the accuracy of the pelvis geometry obtained through SSM-based scaling, a leave-one-out cross-validation analysis was performed. Specifically, the statistical shape model was trained using data from (N-1) participants, and the pelvis geometry of the remaining participant was subsequently reconstructed using the corresponding skin landmark measurements.

To establish the skin landmark dataset, the outer body surface of all 150 subjects was segmented from the corresponding CT scans and reconstructed as skin surface meshes. Bony landmarks (left and right anterior superior iliac spines (ASIS) and posterior superior iliac spines (PSIS)) identified on the mean pelvis mesh (Section 2.3) as vertex indices were expressed across all 150 pelvis meshes. These meshes had been non-rigidly registered using radial basis functions but not rigidly aligned, thereby preserving subject-specific anatomical orientations.

For each pelvis, a local pelvic coordinate system was established from the transferred anatomical landmarks. Skin landmarks were subsequently estimated by projecting rays from the bony landmarks along anatomically relevant directions: anteriorly for the ASIS landmarks and posteriorly for the PSIS landmarks. The first intersection point between each ray and the corresponding skin surface mesh was defined as the skin landmark location. These skin landmarks served as the input measurements for all SSM-based pelvis reconstructions.

The predicted meshes were then compared against the corresponding ground-truth pelvis meshes reconstructed from CT data to evaluate prediction accuracy. Mesh-level geometric accuracy was quantified using the root mean square error (RMSE) between the predicted and ground-truth pelvis geometries, calculated from the node-to-surface distances between each node of the original segmented mesh and the predicted mesh. Joint-level accuracy was assessed by calculating the three-dimensional Euclidean distance errors between the predicted and ground-truth joint centres.

In addition, the accuracy of the SSM-based scaling workflow was compared with two conventional linear scaling approaches by quantifying their respective deviations from the ground-truth meshes:

1) Linear female-pelvis scaling: Linearly scaled mean female pelvis mesh generated from SSM using relative bone length measurements between the mean mesh and the target participant mesh.

2) Linear male-pelvis scaling: Linearly scaled pelvis mesh originated from Raabe’s male OpenSim model [46] using relative bone length measurements between the OpenSim pelvis mesh and the target participant mesh.

For both linear scaling methods, the coordinates of the generic template mesh were scaled isotropically using a scaling factor based on pelvic width, defined as the sum of the ASIS width and PSIS width: Scale Factor = X_participant_/X_generic_, where X = (ASIS_width + PSIS_width).

One-tailed paired t-tests were performed to determine whether the SSM-based scaling workflow significantly reduced reconstruction errors compared with the two linear scaling approaches.

### 2. Accuracy of the pelvis geometry reconstruction

The accuracy of the SSM-based scaling workflow was evaluated using leave-one-out cross-validation against CT-derived ground-truth pelvis meshes and compared with two conventional linear scaling approaches (Table B1).

For pelvis surface geometry, the SSM-based scaling approach achieved the lowest reconstruction error (RMSE: 6.88 ± 2.44 mm), followed by the linear female-pelvis scaling approach (7.13 ± 2.36 mm) and the linear male-pelvis scaling approach (14.38 ± 3.84 mm). The SSM-based approach produced significantly lower errors than the linear male-pelvis scaling approach (*p* < 0.01), while only a small difference was observed relative to the linear female-pelvis scaling approach (*p* = 0.03).

A similar pattern was observed for pelvis-related joint locations. Errors in the predicted sacroiliac joint (SIJ) centres were comparable between the SSM-based scaling approach and the linear female-pelvis scaling approach (all *p* > 0.05), whereas both approaches substantially outperformed the linear male-pelvis scaling approach (all *p* < 0.01).

For the pubic symphysis (PS), L5-S1 joint, and hip joint centres (HJCs), the SSM-based scaling approach consistently achieved the lowest prediction errors. The largest improvement was observed at the PS, where the error increased from 14.58 ± 6.83 mm for the SSM-based approach to 38.91 ± 10.80 mm for the linear male-pelvis scaling approach. Similar statistically significant differences were observed for the L5-S1 joint centre and both HJCs (all *p* < 0.01).

Overall, both SSM-based and female linear scaling substantially improved reconstruction accuracy compared with the generic male pelvis model. The SSM-based approach provided modest but consistent additional improvements, particularly for hip joint centre localisation. However, these improvements were small relative to the overall joint centre errors (~12–15 mm) observed for both approaches.

It should be noted that this validation represents a best-case scenario. Skin landmarks were derived from CT surfaces using ray projection from bony landmarks, eliminating palpation and soft-tissue artefact errors present in experimental motion capture. In addition, bony landmark transfer relied on the SSM correspondence framework, meaning the evaluation is not fully independent of the model structure. Therefore, reported errors should be interpreted as lower-bound estimates of real-world application error.

Table B1. Errors in pelvis geometry reconstruction and pelvis-related joint centre locations across different scaling approaches.

|  | RMSE (mm) | SIJ_l (mm) | SIJ_r (mm) | PS (mm) | L5-S1 (mm) | HJC_l (mm) | HJC_r (mm) |
| --- | --- | --- | --- | --- | --- | --- | --- |
| SSM-based scaling | 6.88 ± 2.44 | 12.15 ± 6.01 | 12.39 ± 6.36 | 14.58 ± 6.83 | 13.58 ± 6.2 | 12.97 ± 6.19 | 13.32 ± 5.87 |
| Linear female-pelvis scaling | 7.13 ± 2.36 | 12.3 ± 6.2 | 12.72 ± 6.44 | 15.68 ± 7.26 | 13.78 ± 6.12 | 14.3 ± 6.35 | 14.82 ± 6.08 |
| *p-value* (female-pelvis) | 0.03 | 0.38 | 0.09 | 0.02 | 0.04 | <0.01 | <0.01 |
| Linear male-pelvis scaling | 14.38 ± 3.84 | 23.16 ± 8.33 | 19.91 ± 7.79 | 38.91 ± 10.8 | 32.43 ± 8.72 | 23.1 ± 8.19 | 23.47 ± 8.79 |
| *p-value* (male-pelvis) | <0.01 | <0.01 | <0.01 | <0.01 | <0.01 | <0.01 | <0.01 |

Values are presented as mean ± standard deviation. Pelvis surface error was quantified using root mean square error (RMSE). Joint centre errors were computed as three-dimensional Euclidean distance (SIJ: sacroiliac joint; PS: pubic symphysis; HJC: hip joint centre). *p-values* were obtained from one-tailed paired t-tests comparing each linear scaling method against the SSM-based scaling approach. p < 0.05 was considered statistically significant.

## Appendix C

### 1. Pelvic joint shear loads in two coordinate systems

Considering that the ‘evaluating’ pelvic joint coordinate system and the ‘anatomically aligned’ coordinate system share the same z-axis, which passes through the joint centre and is orthogonal to the joint’s best-fit plane, the compressive/tensile forces computed from the two coordinate systems are identical. Therefore, in this section, the superior-inferior and anterior-posterior shear forces of the pelvic joints of these two coordinate systems during walking, bilateral standing, and single-leg standing were compared and shown (Figure A1).

Overall, the superior-inferior shear forces of the pelvic joints exhibited largely consistent directions and similar magnitudes across the two coordinate systems (Figure A1). The exception was the pubic joint superior-inferior shear during bilateral standing, where the directions differed; however, the forces were close to zero in this condition (Figure A1). In contrast, the anterior-posterior shear component demonstrated substantially greater sensitivity to the choice of expression coordinate system than the superior-inferior shear components, as substantial differences were observed in anterior-posterior shear forces.

For example, during walking, the peak SIJ superior-inferior shear force differed by 1.3 N/kg between the two joint coordinate systems (24% of the lower value), and the peak pubic joint superior-inferior shear force differed by 0.24 N/kg (10% of the lower value). However, for anterior-posterior shear forces, the discrepancies were much larger: the peak SIJ force differed by 2.93 N/kg (244% of the lower value), and the peak pubic joint force differed by 1.31 N/kg (119% of the lower value).

Figure C1. Comparison of pelvic joint superior-inferior and anterior-posterior shearing between the ‘evaluating’ pelvic joint coordinate system and the ‘anatomically aligned’ coordinate system during walking, bilateral standing, and single-leg standing
